# Extracellular Metabolites of Heterotrophic *Auxenochlorella protothecoides*: A New Source of Bio-Stimulants for Higher Plants

**DOI:** 10.3390/md20090569

**Published:** 2022-09-07

**Authors:** Yujiao Qu, Xinxiang Chen, Beibei Ma, Huachang Zhu, Xuan Zheng, Jiazhen Yu, Qinghui Wu, Rong Li, Ziqiang Wang, Yibo Xiao

**Affiliations:** 1 Protoga Biotechnology Co., Ltd., Zhuhai 519000, China; 2 Microalgae Biosynthesis R&D Center, Research Institute of Tsinghua University in Shenzhen, Shenzhen 518057, China; 3 Sinochem Fertilizer Co., Ltd., Beijing 100069, China

**Keywords:** microalgae, extracellular metabolites, bio-stimulants, higher plant growth

## Abstract

The biodiversity of microalgal species is enormous, and their versatile metabolism produces a wide diversity of compounds that can be used in food, healthcare, and other applications. Microalgae are also a potential source of bio-stimulants that enhance nutrition efficiency, abiotic stress tolerance, and/or crop quality traits. In this study, the extracellular metabolites of *Auxenochlorella protothecoides* (EAp) were prepared using three different culture strategies, and their effects on plant growth were examined. Furthermore, the composition of EAp was analyzed by GC-MS. The elongation of lateral roots and the cold-tolerance of *Arabidopsis thaliana* and *Nicotiana benthamiana* were promoted by EAp. Moreover, EAp from high-cell-density fermentation stimulated the growth of the leafy vegetables *Brassica rapa* and *Lactuca sativa* at dilutions as high as 500- and 1000-fold. Three major groups of compounds were identified by GC-MS, including organic acids or organic acid esters, phenols, and saccharides. Some of these compounds have known plant–stimulating effects, while the rest requires further investigation in the future. Our study demonstrates that EAp is a potential bio-stimulant, while also providing an environmentally friendly and economical microalgae fermentation process.

## 1. Introduction

Modern high yield agricultural crop production largely relies on chemical fertilizers. However, there are increasing concerns related to the impact of chemical fertilizers on soil, water, and air pollution and food safety [1]. As promising additives for chemical fertilizer, bio-stimulants promote plant growth when applied in low quantities, while being non-toxic, non-polluting, and non-hazardous to humans and domestic animals. They play a key role in sustainable intensification through an enhanced efficiency of nutrient uptake and metabolic stimulation [2]. Diverse bio-stimulants from algae, fungi, and bacteria have been used in agriculture to enhance the productivity and quality of products [2].

Microalgae are a diverse group of photosynthetic organisms found in fresh and saline waters and throughout marine systems, including cyanobacteria as well as eukaryotes such as green algae, euglenoids, diatoms and others [3]. They naturally produce many different bioactive compounds, such as proteins, lipids, carotenoids, vitamins, and polysaccharides [4]. Thus, microalgae are a rich source of therapeutic agents for human health [5]. Many studies have shown that microalgal extracts can act as bio-stimulants for higher plants. It was reported that the extracts of *Chlorella vulgaris* and *Scenedesmus quadricauda* exert a positive effect on *Beta vulgaris* germination by increasing the efficiency and regularity of this critical process [6]. The use of filamentous microalgae like *Ulothrix* sp. and *Klebsormidium* sp. as a high-value organic slow-released bio-stimulant resulted in tomatoes with increased carotenoid and sugar levels [7]. Grzesik et al. found that polysaccharides of *Cyanobacteria* and *Chlorella* species can be beneficial to the growth, development, seed germination, and metabolic activity of corn [8]. Two *Chlorella* isolates (MACC-360 and MACC-38) and *Chlamydomonas reinhardtii* cc124 exerted growth stimulative effects on leaf size, biomass accumulation, pigment content, and pod/flower production of *Medicago truncatula* [9].

*Auxenochlorella protothecoides* have been found in a wide variety of environments from fresh water and terrestrial habitats to brackish and marine environments [10]. It is a unicellular eukaryotic microalga that can not only use light and CO_2_ for autotrophic growth, but also use glucose for heterotrophic growth, and is one of the most oleaginous microalgae. *A. protothecoides* can accumulate large amounts of fatty acids, proteins, and lutein, while the cell density in heterotrophic cultivation can reach over 100 times that of autotrophic culture [11]. *A. protothecoides* strain UTEX 2341 is able to grow in both fresh water and artificial seawater mediums [12], this strain was reported by some researchers to shift the fatty acids profile in culture mediums with different salinity [13]. Marine strain LEU27 can accumulate remarkable amount of lutein [14]. *A. protothecoides* cells can be applied in food, health care, biofuels, and other products. The fermentation of *A. protothecoides* on the 60 ton scale has been realized by Xiao et al. for large-scale preparation of microalgal biodiesel [15]. However, the supernatant after fermentation still needs further treatment to meet the discharge standard [16]. *A. protothecoides* secretes various compounds into the extracellular medium to regulate the culture environment [17]. In agriculture, microalgae are mostly used in the form of living cells or cell extracts, while few studies have investigated the effects of extracellular metabolites of heterotrophic *A. protothecoides*.

In order to maximize the utilization of *A. protothecoides*, this study focused on the application of extracellular metabolites of heterotrophic *A. protothecoides* (EAp) to increase plant growth. By testing the functions of EAp as bio-stimulant and identifying its metabolomic profile to explore the special active components, this study provides a theoretical basis for the utilization of extracellular metabolites of microalgae and the rational application of active components in the future.

## 2. Results and Discussion

### 2.1. Effects of EAp on the Growth of Arabidopsis thaliana and Nicotiana benthamiana

**Figure 1:**
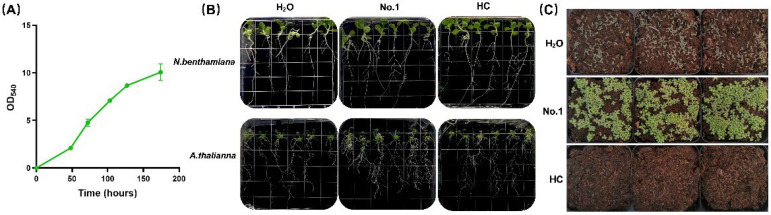
The effect of EAp No.1 on the growth of model plants. (**A**) Growth curve of *A. protothecoides* in HC medium in shake flask from two replicates. (**B**) EAp No. 1 stimulated the development of lateral roots of *A. thaliana* and *N. benthamiana*. Six plants were used in each treatment. (**C**) EAp No. 1 improved the cold tolerance of *A. thaliana* under cold stress. H_2_O: mock treatment with H_2_O; HC: treatment with the heterotrophic culture medium before *A. protothecoides* cultivation.

During heterotrophic culture in shake flasks, *A. protothecoides* can reach an optical density at 540 nm of 10 in 174 h at the late log phase (Figure 1A). In most biotechnological processes, the microalgal cells are economically valuable, while the culture supernatant is often discarded as wastewater. Microalgal cell extracts have been shown to act as plant growth stimulators [3]. It has been shown that microalgal cells secrete a variety of extracellular substances during the cell culture process [18]. Here, we separate the microalgal cells from the culture medium after heterotrophic cultivation and explored the effects of non-cellular components (extracellular metabolites of heterotrophic *A. protothecoides*, EAp) on plant growth. The EAp No. 1 was obtained from the shake flask culture of *A. protothecoides* in standard heterotrophic medium.

The root system is extremely important for plant growth. Roots not only provide mechanical support for the aerial parts of plants, but also absorb water and nutrients from the soil. Moreover, roots also play a role in sensing biotic and abiotic stresses [19]. The addition of 1000-fold diluted EAp No.1 to MS solid medium significantly promoted the elongation of lateral roots of *N. benthamiana* and *A. thaliana* (Figure 1B). Fang et al. found that some yeasts secrete IAA to promote lateral root growth [20]. The fungus *Trichoderma* sp. was found to promote lateral root growth as a bio-stimulant [21]. Researchers also found that the microalga *Acutodesmus dimorphus* and six seaweed extracts could increase the number of lateral roots in mung beans [22,23]. Although there was no obvious effect of EAp No. 1 on the total length of the main root and the number of lateral roots, the promoted growth of lateral roots can significantly improve the ability of plants to acquire water and nutrients, potentially increasing the plant biomass and crop yields.

Cold stress, which reduces crop yield and quality, is one of the major abiotic stresses that limits crop productivity and agricultural sustainability worldwide. Studies have shown that low temperatures can reduce root branching and surface area [24]. After finding that EAp No. 1 treatment increases the root lateral length (Figure 1B), it was pertinent to investigate if this effect would protect plants exposed to cold stress. *A. thaliana* were grown in soil outdoors when the lowest night temperature was 13 °C. After 17 days, the growth of control plant seedlings was stunted and the leaves exhibited a purple discoloration, a sign of stress. When treated with EAp No. 1, the leaves of *A. thaliana* seedlings remained green, and the growth rate was significantly higher than that of control seedlings treated with water. Moreover, though the HC could also stimulate the growth of lateral roots, the seedlings irrigated with heterotrophic culture medium (HC) without algal cultivation died two days after germination (Figure 1C). Importantly, this result indicates that the plant stimulation effect is due to extracellular metabolites of *A. protothecoides* instead of the culture medium itself. As a result, the HC control was excluded from further analysis. Studies have shown that macroalgae are rich in polysaccharides, phenolics, fatty acids, vitamins, osmolytes, phytohormones, and hormone-like compounds, which can improve the abiotic stress responses of higher plants [25]. Similarly, metabolites of microalgae were found to alleviate salt and drought stress in higher plants [26]. As a result, we speculated that EAp could improve the performance of higher plants under abiotic stress.

### 2.2. EAp Treatment Affected the Growth and Nutritional Value of Brassica chinensis

Leaf vegetables are an important part of the human diet that offer many health benefits. They are low in sodium and calories, while being rich in minerals, folic acid, and fibers, as well as natural antioxidants such as polyphenols, flavonoids, vitamins, and carotenoids [27]. Here we tested the effect of EAp No. 1 on the growth and nutritional composition of the leaf vegetable *Brassica chinensis*. Two different amounts of EAp No. 1 were applied to 25-day-old plants when the biomass increases dramatically and requires large amount of fertilizer input. The amount of EAp No. 1 in the treatment was 45 mL and 90 mL for each pot, respectively.

**Figure 2:**
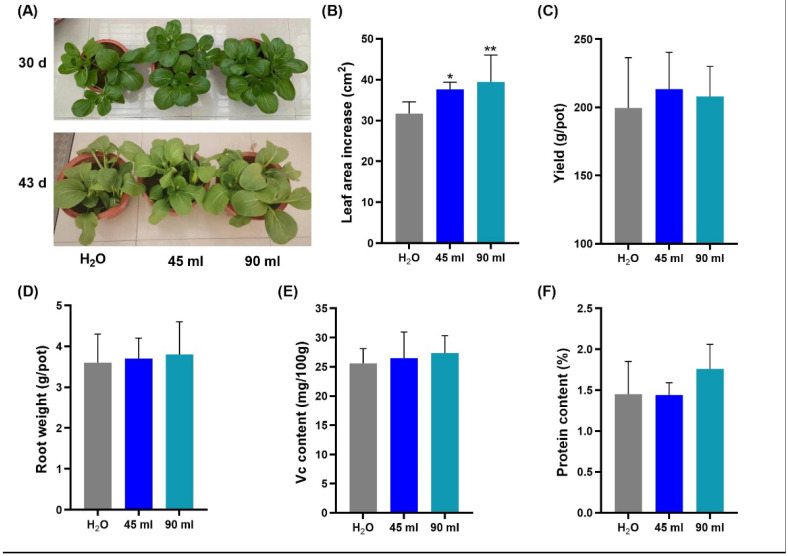
The effects of EAp No. 1 on the growth and nutritional value of *B. chinensis*. (**A**) Growth effect of EAp No. 1 on *B. chinensis*, on the 30th and 43rd days, 5 and 18 days after treatment, respectively. (**B**–**F**) The effect of EAp treatment on the leaf area increase, total biomass, root weight, vitamin C content (ascorbic acid, mg/100 g), and protein content. 45 mL and 90 mL of EAp No. 1 was irrigated in the soil, respectively. H_2_O: Mock treatment with H_2_O. Columns denoted by one or two asterisk(s) are significantly different from the water control at *p* < 0.05 and *p <* 0.01, respectively. Values represent average (*n* = 7); bars represent standard error.

The growth of the plants after 5 days of treatment was obviously better than that of the control (Figure 2A, upper panel) and the non-destructive measurements were done on this day. The leaf area increase was 26.8 and 29.3% larger for the two treatments compared to the control, respectively. The appearance of the plants was similar when harvested after 18 days after treatment (Figure 2A, lower panel). Importantly, the yield of the 45 mL treated plant was 7% higher. As the roots largely influence plant growth, we measured the fresh weight of the roots and found that the treatment with EAp No. 1 resulted in increases of 4.2 and 6.7% of *A. thaliana* and *N. benthamiana*, respectively. This was in agreement with Figure 1B, which shows that *A. thaliana* and *N. benthamiana* grown on plates with EAp had longer lateral roots.

Nutritive value is an essential quality index for vegetables. The vitamin C (ascorbic acid) content of *B. chinensis* under the two treatments was increased by 3.4 and 6.9% in the 45 mL and 90 mL EAp No. 1 treatment compared to H_2_O, while the protein content was increased by 21.4% in the 90 mL EAp No. 1 treatment compared to H_2_O. Leaf area is directly related to photosynthesis and affects crop yield [28]. This study found that the application of EAp No. 1 can increase the yield of leafy vegetables, while increasing vitamin C and protein content. Vitamin C is an important multifunctional metabolite. It is a co-substrate for a large number of enzymes, and influences many metabolic reactions in the cell [29]. Humans must acquire vitamin C regularly from dietary sources such as leafy vegetables [30]. This metabolite is one of the most abundant in green leaves. Therefore, EAp No. 1 can improve the nutritive value of leaves and could be used in the production of high-quality vegetables.

### 2.3. EAp Varies According to Cultivation Strategy

Increasing the concentration of the bio-stimulants offers several advantages, such as smaller storage space and less labor in distribution. In this study, we made efforts to concentrate the EAp by applying different cultivation strategies and testing the effect on plant growth. It is well known that the nutritional value of microalgae varies according to the composition of the culture medium. The nitrogen content in the heterotrophic medium greatly influences the lipid and protein content of *A. protothecoides* [31], and the extracellular metabolites would also be influenced as a consequence. Here, we tested the effect on plant growth of extracellular metabolites of *A. protothecoides* cultured in medium with two nitrogen levels in shake flasks. The optical density at 540 nm of *A. protothecoides* in high nitrogen medium (HN, EAp No. 2) was 9.0, and the protein content was 52.6%, which was much higher than that of *A. protothecoides* in low-nitrogen medium (HC, EAp No. 1) with a protein content of 10.3%.

**Figure 3:**
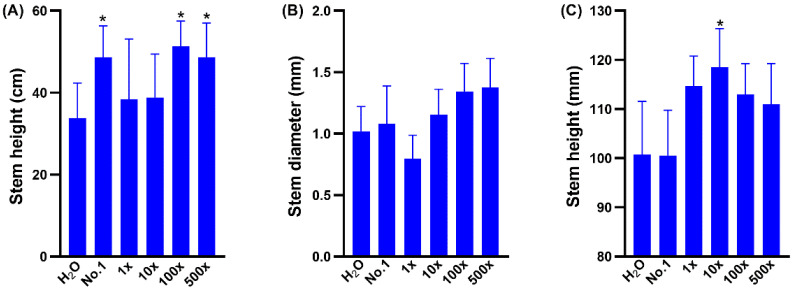
The growth effect of EAp No. 1 and series diluted EAp No. 2 on higher plants. The effect of EAps on (**A**) the stem height of *Cucumis sativus* and (**B**) the stem diameter of *Lycopersicon esculentum*, as well as (**C**) the stem height of *Lycopersicon esculentum.* H_2_O: Mock treatment with H_2_O. No. 1: EAp No. 1 treatment. 1×, 10×, 100× and 500× indicate 1-, 10-, 100- and 500- fold diluted EAp No. 2 treatment, respectively. Columns denoted by one asterisk is significantly different from the water control at *p* < 0.05, respectively. Values represent average (*n* = 5); bars represent standard error.

As it has been reported that most of the bio-stimulants and fertilizers affect the plant growth at a concentration-dependent manner, we therefore carried out a series of dilution experiments and found that the stem diameter of *L. esculentum* is smaller than the control (Figure 3B). In contrast, the stem height of *Cucumis sativus* treated with EAp No. 1 was 31% higher than that of the control. Notably, EAp No. 2 produced a similar growth promoting effect at 100-fold and 500-fold dilution. Treatment with the 100-fold dilution of EAp No. 2 increased the stem diameter and stem height of *L. esculentum* by 32 and 12% compared with the control, respectively. Treatment with the 500-fold dilution of EAp No. 2, increased the *L. esculentum* stem diameter and height by 35 and 10% compared with the control, respectively. These results indicate that the functional components of EAp accumulate to a higher level in high-nitrogen medium, and thus can be used as a concentrated bio-stimulant.

**Figure 4:**
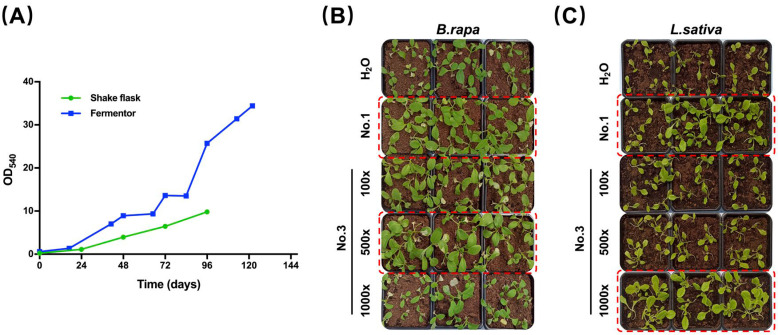
The growth-promoting effect of EAp No. 3 on two leaf vegetables. (**A**) Growth curves of *A. protothecoides* in shake flask and fermenter culture. The growth-promoting effect of a series of diluted EAp No. 3 preparations on (**B**) *Brassica rapa* and (**C**) *Lactuca sativa*. H_2_O: Mock treatment with H_2_O. No. 1: EAp No. 1 treatment. 100×, 500× and 1000× indicate 100-, 500- and 1000-fold diluted EAp No. 3 treatment, respectively.

Brown macroalgae are the most commonly used algae in agriculture, and dozens of commercial products have been developed based on their biomass [32]. *Ascophyllum nodosum* seaweed extracts can activate the immune response genes and inhibit the growth of multiple bacterial pathogens in *A. thaliana* [33]. Similarly, brown algae extract was shown to trigger the defense response of tomato by inducing phenylalanine ammonia-lyase activity and phenolic compounds [34]. Jimenez et. al. found some of the macroalgae extract have the antibacterial function from a survey of nine macroalgae species collected from four different year periods [35]. However, macroalgae are mostly harvested from the sea, and their composition varies with tissue age, environmental conditions, nutrient availability, and harvesting time, which hinders the standardization of raw material quality [35]. Ideally, a successful bio-stimulant should not only be sustainable and effective, but also based on organic by-products and able to favor the closure of the nutrient loops in agriculture [36]. Industrialized production of *A. protothecoides* in constant fed-batch fermentation has higher production efficiency, and can achieve far higher cell densities than shake-flask culture (Figure 4A). The highest cell density of fermentation after 122 h can reach 34 (OD_540_), and the dry weight reaches 60.1 g/L, which is more than three times that of shake flask culture. Correspondingly, we speculated that the content of the extracellular components that function in plant growth stimulation is also higher.

After 122 h of fed-batch fermentation, the culture was centrifuged to remove the cells, and the supernatant was designated as EAp No. 3. We have found that EAp No. 1 promotes cold tolerance and stimulates plant growth. EAp No. 3 is considered as a concentrated version of EAp No. 1, and we would like to find out the dilution factor for EAp No. 3, which has a growth-promoting effect similar to EAp No.1 by series dilution experiments on two leaf vegetable species. The effects of EAp No. 3 on plant growth were tested after 100-, 500- and 1000-fold dilution (Figure 4B,C). EAp No. 3 exhibited a growth-promoting effect similar to that of EAp No. 1 at 500-fold and 1000-fold dilution for *Brassica rapa* and *Lactuca sativa*, respectively (Figure 4B,C, dashed-line boxed). These results indicate that the supernatant from *A. protothecoides* fermentation can be used as a concentrated bio-stimulant. As the cultivation strategy influences the EAp, the progress of the fermentation process also affects the quality of bio-stimulants. The reuse of the culture medium can greatly ease the pressure of wastewater production, making the fermentation of *A. protothecoides* more environmentally friendly and economical. More importantly, the stringent process control during the fed-batch fermentation ensures the quantity and quality of microalgal cell product, while at the same time guaranteeing the quantity and quality of EAp from the same process.

### 2.4. Chemical Composition of EAp

Since EAp was found to promote the growth or stress resistance of higher plants, we further analyzed the active components that play a key role in the effects of EAp. For EAp No. 1 and No. 2, we detected common components also found in conventional plant fertilizer, such as N, P, K, as well as some trace elements such as Fe, Mg, etc., and free amino acids (Table 1). The table shows that the content of organic matter in EAp No. 1 and No. 2 was 0.12 and 1.18%, respectively. The total nitrogen content was 0.42 and 0.31%, respectively. There was no detectable boron or insoluble substances in the two EAp preparations. Usually, the nitrogen content of chemical fertilizer is no less than 15%, and the organic matter content of organic fertilizer is usually more than 20% [37]. The content of organic matter and nitrogen in EAp is therefore much lower than that of common fertilizer and cannot explain its effects. Moreover, the content of trace elements (Fe, Zn, B) in EAp was also much lower than in common trace element fertilizer. Compared with common fertilizer, the amount of these components in EAp are extremely low and can hardly play a role in promoting plant growth or stress resistance. To explore substances that may play a key role as bio-stimulants, we considered identifying other compounds in EAp that are not commonly used in fertilizer.

**Figure 5:**
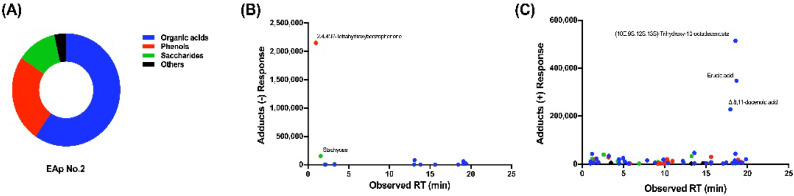
EAp No.2 components from shake flask with HN medium identified by GC-MS-based metabolite profiling analysis. (**A**) Distribution of component types in EAp No. 2. Abundance of components in EAp No. 2 identified by (**B**) negative and (**C**) positive charge mass spectrometry. The color code used in (**B**,**C**) is consistent with (**A**).

We performed GC-MS identification experiments on EAp No. 2. After library searching, 14 compounds were identified in EAp No. 2 by negative charge mass spectrometry, as well as 70 compounds by positive charge mass spectrometry (Tables S1 and S2). The compounds identified in EAp No. 2 are 50 organic acids or organic acid esters, 21 phenols, and 13 saccharides or 3 other compounds (Figure 5A).

The most abundant compounds identified in EAp were organic acids and organic acid esters (Figure 5C; Tables S1 and S2), among which the top five were (10E, 9S, 12S, 13S)-trihydroxy-10-octadecenoate, erucic acid, Δ8,11-docenoic acid, celestial acid, ricinoleic acid, and linolenic acid. Plants themselves secrete organic acids in response to certain nutrient deficiencies, including monocarboxylic acids (acetic, formic, glycolic, and lactic), dicarboxylic acids (malic, oxalic, and succinic), and tricarboxylic organic acids (citric and trans-aconitic acid) [38]. Organic acids may play an important role in the nutrient uptake of higher plants. In case of nutrient stress, the release of organic acids may promote the dissolution of metal oxides in soil, thus increasing the availability of trace elements such as iron, zinc, and copper [39]. The organic acids and organic acid esters in EAp may therefore help plants absorb nutrients and reduce carbon loss.

Phenols are aromatic compounds with one or more hydroxyl groups, which play different roles according to their chemical structures, including antibacterial or antioxidant effects, strengthening the cell wall, preventing water loss, or acting as signaling molecules [40]. Under the influence of abiotic stress, the synthesis of phenolic compounds such as flavonoids in plants increases in response to environmental constraints [41]. At the same time, phenolics play a key role in developmental processes such as cell division, hormonal regulation, photosynthetic activity, nutrient mineralization, and reproduction [40,42]. Various phenols secreted by *A. protothecoides* such as 2,4,4′,6′-tetrahydroxybenzophenone, 2′-acetyl cimicifugin, octahydrocurcumin, 6-zinedione, and dozens of other phenols have been identified. There are few studies on the bio-stimulatory effects of these phenolic compounds, and this study provides a basis for broader research and development prospects.

Glycosides were also relatively abundant in EAp, including 3,4,5-Trimethoxyphenyl-1-O-[β-D-furoseyl-(1→6)]-β-D-glucopyranoside, 4-Methoxybenzaldehyde-2-O-[β-D-xylose(1→6)β-D-glucose], 3,4,5-trimethoxyphenyl-1-O-β-D-glucopyranoside, N-hexyl-β-D-glucoside, 4-hydroxyphenylethanol-4-O-β-D-glucopyranoside, curcumone glucoside, 2,3-butanediol monoglucoside, methyl-β-D-fructopyranoside, ethyl galactoside, and citrus glycoside C. In addition, there were also some other saccharides such as 1F-Fructofuranosylnystose, stachyose, mannose, sucrose, and isomaltose. Bournonville et al. reported that Arabidopsis treated with strawberry acyl glycosides exhibited stronger resistance against both bacterial and fungal pathogens [43]. There are many studies on the regulatory effects of microalgal polysaccharides on plants. Microalgal polysaccharides can increase the ascorbate content of plants as well as the activities of NADPH-synthesizing enzymes and ascorbate peroxidase, which have effects on photosynthesis, cell division, and abiotic stress tolerance [44]. Chlorella-derived polysaccharides have bio-stimulatory effects that improve plant growth, pigment content, and fresh biomass [45]. Glycosides have a wide range of biological effects, including antifungal and antibiotic effects, but also negative side-effects such as cytotoxicity, neurotoxicity, and phytotoxicity [43,46]. They are of great importance to the metabolism of various organisms, but much remains to be elucidated in terms of their roles and properties.

## 3. Materials and Methods

### 3.1. Microalgal Cultivation and EAp Preparation

The microalga strain used in this study was *Auxenochlorella protothecoides* 0710 from Prof. Qingyu Wu at Tsinghua University, Beijing, China. The *A. protothecoides* heterotrophic culture medium (HC medium) contained: K_2_HPO_4_ 0.3 g/L, KH_2_PO_4_ 0.7 g/L, MgSO_4_ 0.3 g/L, FeSO_4_·7H_2_O 3 mg/L, V_B1_ 10 μg/L, A5 trace mineral solution 1 mL/L, glycine 4 g/L, glucose 30 g/L, and yeast extract 1 g/L. The recipe of A5 trace mineral solution consists of H_3_BO_3_ 286 mg/L, MnSO_4_·7H_2_O 250 mg/L, ZnSO_4_·7H_2_O 22.2 mg/L, CuSO_4_·5H_2_O 7.9 mg/L, and Na_2_MoO_4_·2H_2_O 2.1 mg/L. For the seed culture, a single colony of *A. protothecoides* from solid HC medium was used to inoculate 20 mL of liquid HC culture medium and cultured for 4 days till the optical density at 540 nm (OD_540_) reaches 8. The seed cultures were conducted under constant orbital shaking at 220 rpm at 28 °C in darkness. The heterotrophic high-nitrogen culture medium (HN medium) was the same as the HC medium, except that the glycine concentration was 5 g/L and yeast extract concentration was 2 g/L.

#### 3.1.1. Shake-Flask Culture

The seed culture was used to inoculate 1 L of the same medium to an initial OD_540_ of 0.2, and the culture was conducted in a shaker in darkness at 220 rpm and 28 °C. The culture of *A. protothecoides* was collected when the remaining glucose concentration was less than 5 g/L, at which point the OD_540_ value usually reaches 8.8–9.0. The culture was centrifuged at 5000× *g* for 2 min to precipitate the cells. The supernatant was autoclaved at 108 °C for 30 min and used as EAp. All the EAp samples were prepared the same way unless stated otherwise. EAp No. 1 was from shake flask cultures in the HC medium. EAp No. 2 was from shake flask cultures in the HN medium.

#### 3.1.2. Fermentor Culture

Fermentation of *A. protothecoides* was conducted with a 5 L fermentor (model: GBJS-5L-AUTOBIO, Zhenjiangdongfang, Jiangsu, China). Shake flask culture was used as seed for fermentation. The initial fermentation conditions were as follows: temperature 28 ± 0.5 °C, pH 6.3, dissolved oxygen concentration (pO2) 100%, and agitation speed 300 rpm. The concentrated glucose and yeast extract solution were batch-fed, and the pH was controlled to 6.5 by NaOH. During fermentation, the concentration of sucrose was controlled in the range of 8–25 g/L by batch-feeding manually, while all other parameters were controlled automatically. The details of the culture conditions were as described previously [16]. The pO2 was kept above 20% by modulating agitation speed and airflow [47]. After the fermentation, the culture was centrifuged and autoclaved the same way as the shake flask culture, resulting in EAp No. 3.

### 3.2. Root Development in Petri Dish Culture

The root development experiment was performed on a solid MS medium with 30 g/L sucrose and 5% agar (BOSF MP0202). 1000-fold diluted EAp No.1 was added to the MS medium. MS medium and MS medium with the 1000-fold diluted HC medium were used as mock control and negative control, respectively.

*A. thaliana* and *N. benthamiana* seeds were germinated in the MS solid medium, and were subsequently transferred to an MS solid medium containing different EAps, with six seedlings in the same horizontal position in each plate. The plate was positioned at a near- vertical angle to facilitate downward root growth. The seedlings were cultivated at 25 °C, with 2500 lux light intensity and 14/10 h light/dark cycle. The pictures of roots were taken when the roots almost reached the bottom of the petri dish. The root length was measured by a ruler.

### 3.3. Plant Growth

The soil (K413, Klasmann, Geeste, Germany, without fertilizer) was soaked with water and placed in growing pots. For the cold stress experiment, the seeds of *A. thaliana*, *N. benthamiana*, and *L. esculentum* were sowed evenly in the soil. Each pot contained 1 kg of soil, and was irrigated with 7 mL of EAp 72 h post imbibition. The EAps were used without dilution unless stated otherwise. The plants are watered regularly afterwards, and kept outdoors with a temperature range of 13 °C to 25 °C and 11 h daylight. For the dilution series experiment, five plants of *L. esculentum* and *C. sativus* were planted in soil after germination between two pieces of wetted paper. The EAp No. 2 was diluted to 10-, 100- and 500-fold. The treatments with series diluted EAp No. 2 were applied after leaves emerged from the soil. Stem height and diameter were measured 50 days after planting by a ruler and vernier caliper, respectively. Nine seedlings of *B. rapa* and *L. sativa* were grown in each pot with 2 kg of soil, and each plant was irrigated with 1 mL of 100-, 500- and 1000-folded diluted EAp. No. 3. after leaves emerged from the soil.

For the growth and nutrition value experiment, three *B.* *chinensis* seedlings were grown in each pot with 9 kg of soil. Each pot was irrigated with 45 or 90 mL of EAp 25 days after imbibition. Water irrigation was used as mock control and each treatment was conducted in seven replicates on day 25. New leaves of a similar size were marked on each plant before the treatment. The leaf area of the marked leaf was measured on the 25th, 30th, and 43rd day, that is, before treatment, as well as 5 and 18 days after treatment. The contents of chlorophyll, protein, and vitamin C, as well as the leaf area, biomass and fresh root weight, were measured on the 43rd day when the plants were harvested. The leaf area was measured using a leaf area meter (LI-3100, LI-COR, Lincoln, NE, USA). The protein content was measured using the Bradford method. In brief, the sample was digested with nitric acid, after which a chromogenic agent was added, followed by measuring the absorbance at 400 nm. Vitamin C was detected by LC-MS on a C18 column. The final yield and root fresh weight were weighed by a balance.

### 3.4. EAp Composition Analysis

The composition of EAp was examined using GC-MS. Chromatography was performed on an Agilent 8890-7010B GC-MS solution system (Agilent, Santa Clara, CA, USA) equipped with a HP-5ms capillary column (30 m × 250 μm i.d., 0.25-μm film thickness; Agilent J&W Scientific, Folsom, CA, USA). Samples (1 μL) were injected with a split ratio of 1:1 by the Agilent autoinjector. Helium was used as the carrier gas at a constant flow rate of 1 mL/min. The injector temperature was set at 300 °C. The GC oven temperature was heated to 60 °C for 1 min, raised to 80 °C at a rate of 15 °C/min, raised to 260 °C at a rate of 10 °C/min, raised to 280 °C at a rate of 8 °C/min and then maintained at 325 °C for 5 min. The ion source and ion source surface temperatures were set to 240 °C and 280 °C, respectively. Electron impact ionization (70 eV) at full scan mode (*m*/*z* 50–800) at a rate of 20 scans/s was used. The acceleration voltage was turned on after a solvent delay of 4 min. Ribitol served as an internal standard to monitor batch reproducibility and to correct for minor variations that occurred during sample preparation and analysis. GC-MS Real Time Analysis software (Agilent, Santa Clara, CA, USA) was used to acquire mass spectrometric data. Mass spectra of all detected compounds were compared with spectra in the NIST library 2.4, the in-house mass spectra library database established by Umeå Plant Science Center. The nitrogen content was measured using a Kjeldahl determination instrument (Hanon, model K9840). The phosphate content was measured using the quinoline phosphomolybdate gravimetric method, and GC-MS was performed as described previously [48,49].

### 3.5. Statistic Analysis

The measurements of growth parameters were subjected to one-way analysis of variance (ANOVA) to test difference among means via GraphPad Prism software (Version 8.3.0, San Diego, CA, USA). A post hoc *t*-test was used for the analysis of significant difference between treatments. The level of significance was set at *p <* 0.05.

## 4. Conclusions

The extracellular metabolites of *Auxenochlorella protothecoides* (EAp) would normally be discarded as waste, but in this study, we found that EAp has a beneficial effect on plants, promoting growth and abiotic stress tolerance. Three versions of EAp were prepared using different cultivation strategies, and the EAp from fed-batch fermentation promoted plant growth even at high dilutions, providing a concentrated bio-stimulant. The composition of EAp was also investigated and three major groups of compounds were found, some of which have been shown to play a role in regulating plant growth, while most of them have not yet been studied. Consequently, we propose that EAp can be used as a more economical, more sustainable, and green bio-stimulant additive in agriculture. The various compounds found in this study could be candidates for microalgae-derived plant bio-stimulants. The detailed quantitative studies of the effects of individual compounds derived from EAp on different plants and their economic traits may lead to the discovery of bio-stimulants with known function and composition.

## Figures and Tables

**Table 1:** Macro- and micro-elemental composition of EAp No. 1 and No. 2 (*w*/*w*). OM: organic matter.

Sample	OM	N	P	K	Ca	Mg	Fe	Mn	Zn	AA
**No. 1**	0.12	0.42	0.015	0.006	0.003	0.001	0.001	-	-	-
**No. 2**	1.18	0.31	0.05	0.044	0.037	0.016	0.0007	0.0001	0.0002	0.102

## Data Availability

Not applicable.

## Supplementary Materials

The following supporting information can be downloaded at: https://www.mdpi.com/article/10.3390/md20090569/s1, Table S1: Components of EAp No. 2 identified by negative charge mass spectrometry; Table S2: Components of EAp No. 2 identified by positive charge mass spectrometry; Table S3: Summery of plants and EAp treatments.
